# Incorporating Methyl and Phenyl Substituted Stannylene Units into Oligosilanes. The Influence on Optical Absorption Properties

**DOI:** 10.3390/molecules22122212

**Published:** 2017-12-12

**Authors:** Filippo Stella, Christoph Marschner, Judith Baumgartner

**Affiliations:** 1 Institute for Inorganic Chemistry, Graz University of Technology, Stremayrgasse 9, 8010 Graz, Austria; filippo.stella@unipd.it; 2 Institute for Chemistry, Karl-Franzens-University Graz, Stremayrgasse 9, 8010 Graz, Austria

**Keywords:** stannaoligosilanes, σ-bond electron delocalization, UV-spectroscopy, single crystal diffraction analysis

## Abstract

Molecules containing catenated heavy group 14 atoms are known to exhibit the interesting property of σ-bond electron delocalization. While this is well studied for oligo- and polysilanes the current paper addresses the UV-absorption properties of small tin containing oligosilanes in order to evaluate the effects of Sn–Si and Sn–Sn bonds as well as the results of substituent exchange from methyl to phenyl groups. The new stannasilanes were compared to previously investigated oligosilanes of equal chain lengths and substituent pattern. Replacing the central SiMe_2_ group in a pentasilane by a SnMe_2_ unit caused a bathochromic shift of the low-energy band (λ_max_ = 260 nm) of 14 nm in the UV spectrum. If, instead of a SnMe_2,_ a SnPh_2_ unit is incorporated, the bathochromic shift of 33 nm is substantially larger. Keeping the SnMe_2_ unit and replacing the two central silicon with tin atoms causes shift of the respective band (λ = 286 nm) some 26 nm to the red. A similar approach for hexasilanes where the model oligosilane [(Me_3_Si)_3_Si]_2_(SiMe_2_)_2_ (λ_max_ = 253 nm) was modified in a way that the central tetramethyldisilanylene unit was exchanged for a tetraphenyldistannanylene caused a 50 nm bathochromic shift to a low-energy band with λ_max_ = 303 nm.

## 1. Introduction

It is well known that oligo- and polymeric chains of silicon, germanium, and tin atoms possess the unusual feature of σ-bond electron delocalization [1]. This property reflects the smaller HOMO-LUMO gaps of higher elements and in a molecular way resembles the semiconducting nature of the higher group 14 elements and is best known for polysilanes [2].

Despite the fact that σ-bond electron delocalization seems to be an exotic property it is actually not so much different from the much more familiar concept of π-bond electron delocalization. Both types of delocalization rely on two important prerequisites. The first requirement is an electronic one and it says that HOMO-LUMO gaps need to be reasonably small. This requirement is not met for alkanes which is the reason that σ-bond electron delocalization is very much restricted to compounds containing σ-bonds between heavier elements. The second prerequisite is a spatial one and it requires the involved orbitals to be in plane to allow extended orbital overlap over the involved atoms along the chain. For the π-bond electron delocalization this means all delocalized bonds are in one plane. A similar overlap for σ-bond electron delocalization would require an *all-trans*-conformation of the involved chain. As catenated heavier elements usually bear comparatively large substituents there are steric 1,2- and 1,3-interactions between these substituent, thereby causing a deviation from *all-trans*- to a *transoid* conformation.

While the phenomenon of σ-bond electron delocalization has first been recognized by the research groups of Gilman and Kumada [3,4,5], the theoretical foundations of the effect have been studied in some detail by Michl and others [1,6,7,8]. Eventually, Tsuji and Tamao proved the concept of conformational dependence of σ-bond electron delocalization in oligosilanes, preparing a number of conformationally constrained molecules [9,10,11,12,13].

Our group and others have shown that oligosilanes with large end groups exhibit a preference to acquire a transoid conformation as long as the end groups are not too far apart from each other [14,15,16,17,18,19,20]. In a recent study we reported on the influence of the formal replacement of silicon atoms in oligosilanes by germanium atoms on the σ-electron delocalization properties, which was found to be rather negligible [21]. However, related studies by Weinert and co-workers on the optical absorption properties on oligogermanes [22,23,24,25,26,27,28,29,30,31] concentrated mainly on phenylated molecules which were found to exhibit bathochromic shifted low-energy bands compared to our methylated oligosilanes of the same chain lengths. This is not unexpected, as the introduction of more electronegative substituents is known to cause absorption bands of oligosilanes to shift to longer wavelengths.

The current account is concerned with the influence of the formal exchange of silicon by tin atoms and of methyl groups by phenyl substituents on the optical absorption properties, which reflect the extent of σ-bond electron delocalization.

## 2. Results and Discussion

### 2.1. Synthesis

Over the last 20 years, our group has prepared many different oligosilanes. The key synthetic advancement that allowed for the preparation of structurally reasonably complex molecules was the discovery of the facile silanide formation accomplished by silyl group abstraction with potassium alkoxides [32]. The potassium silanides thus obtained proved to be ideal building blocks, which could be reacted with silyl halides of triflates to a large variety of oligosilanes. Subsequent application of the silyl group abstraction method to silylated germanes [33] and stannanes [34] provides access to silylated germanides and stannides. Synthesis of organostannanes with attached oligosilanyl groups as required for the current study therefore could be achieved in a fairly straightforward way.

**Scheme 1:**
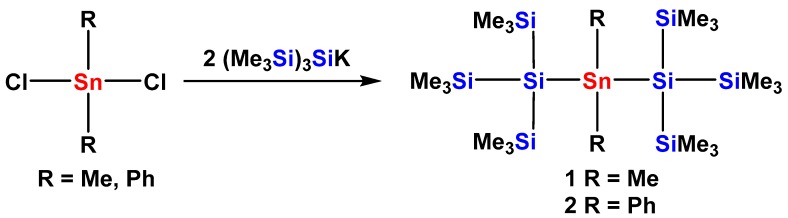
Synthesis of bis[tris(trimethylsilyl)silylated] stannanes **1** and **2**.

Reactions of tris(trimethylsilyl)silyl potassium [35,36] with dimethyldichlorostannane or diphenyldichlorostannane provided bis[tris(trimethylsilyl)silyl]dimethylstannane (**1**) [37] and bis[tris(trimethylsilyl)silyl]diphenylstannane (**2**) in 86% and 61% yield, respectively (Scheme 1).

The NMR spectroscopic properties observed, and in particular the ^29^Si and ^119^Sn chemical shifts of 1 [37] and 2 (Table 1), are quite similar and completely within the range of what to expect [38,39].

**Figure 1:**
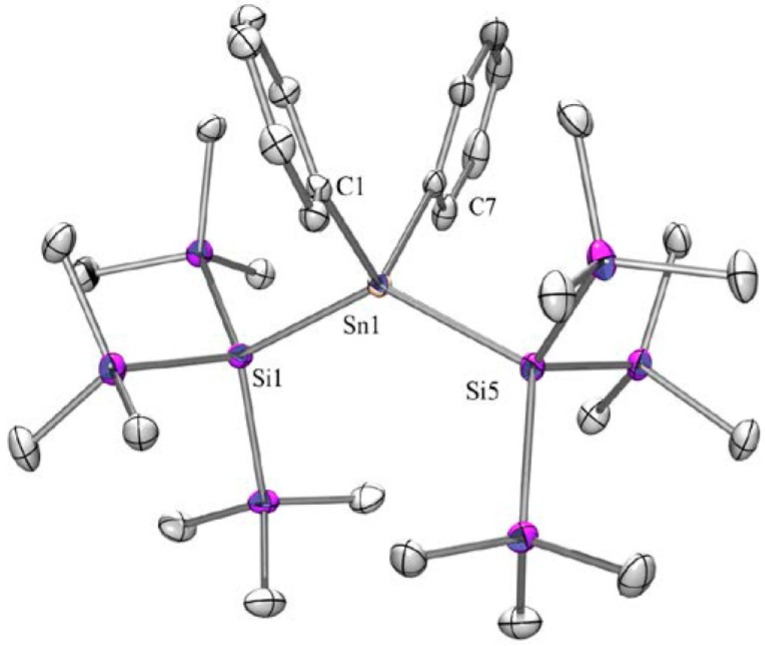
Molecular structure of **2** (thermal ellipsoid plot drawn at the 30% probability level). All hydrogen atoms are not shown for clarity (bond lengths in Å, angles in °). Sn(1)–C(7) 2.1603(19), Sn(1)–C(1) 2.1609(19), Sn(1)–Si(1) 2.6071(7), Sn(1)–Si(5) 2.6099(8), Si(1)–Si(2) 2.3747(9), Si(2)–C(13) 1.872(2), Si(1)–Sn(1)–Si(5) 132.507(17).

Single crystals of compound **2** were subjected to XRD analysis (Figure 1) showing **2** to crystallize in the monoclinic space group *P*2_1_/*n*. Structural parameters include Sn-Si bond distances of 2.6071(7) and 2.6099(8) Å and an Si–Sn–Si bond angle of 132.507(17)°. While a number of tin compounds with two attached tris(trimethylsilyl)silyl groups are known, most of the structurally characterized examples feature divalent tin, where the Sn–Si bonds are significantly elongated to values above 2.65 Å and the Si–Sn–Si bond angles are smaller than 115° [40,41,42,43]. Nevertheless dichlorostannane [(Me_3_Si)_3_Si]_2_SnCl_2_ [37] was found to exhibit quite similar Si-Sn distances of 2.604(3) Å and the Si-Sn-Si angle of 142.5(1)° was even somewhat narrower than in **2**. For the structurally related cyclic compound (Me_2_Si)_2_[(Si(SiMe_3_)_2_]_2_SnPh_2_ [44] the Si–Sn distances amount to 2.594(4) and 2.620(4) Å. For the related compounds (Me_3_Si)_3_SiSn*^i^*Pr_3_ with only one tris(trimethylsilyl)silyl group and three isopropyl groups on tin and hexakis(trimethylsilyl)distannane even shorter Si-Sn distance of 2.573(4) and 2.5707(17) Å, respectively, were reported [45]. Of particular interest for the σ-bond delocalization are the conformational properties, which are reflected by the dihedral angles. As listed in Table 2, the two relevant dihedral angles of **2** are exhibiting a deviant conformational preference, which is likely caused by steric interactions of the phenyl groups with trimethylsilyl groups.

**Scheme 2:**
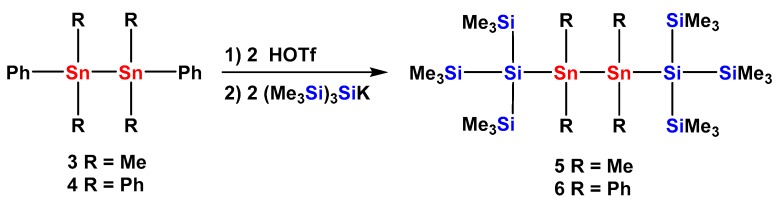
Synthesis of bis[tris(trimethylsilyl)silylated]distannanes **5** and **6**.

Starting out from 1,2-diphenylated distannanes **3** [46,47] and **4**, the respective triflates were obtained by treatment with two equiv triflic acid and treated further on with two equiv tris(trimethylsilyl)silyl potassium. Tris(trimethylsilyl)silylated distannanes **5** and **6** were formed in 48% and 33% yields (Scheme 2). In addition to distannane **6** a few crystals of a tris(trimethylsilyl)silylated tristannane (**6a**) were obtained. Although it was not possible to subject **6a** to NMR spectroscopic characterization, the few crystals obtained permitted crystallographic analysis (*vide infra*).

**Figure 2:**
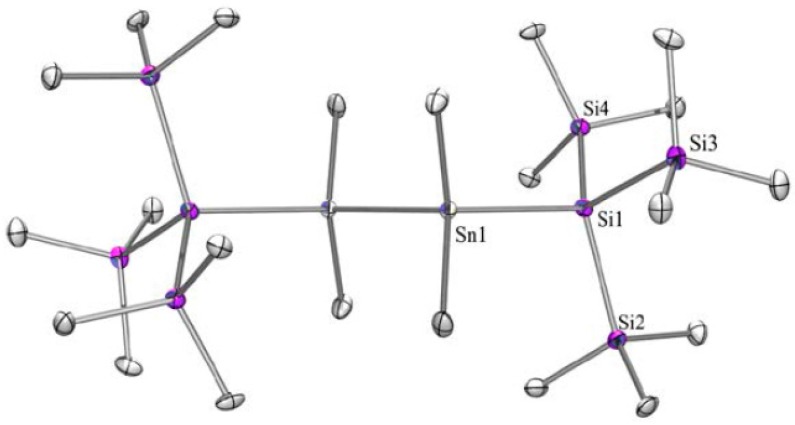
Molecular structure of **5** (thermal ellipsoid plot drawn at the 30% probability level). Non-labeled atoms are generated by symmetry operations. All hydrogen atoms are not shown for clarity (bond lengths in Å, angles in °). Sn(1)–C(11) 2.182(9), Sn(1)–Si(1) 2.579(3), Sn(1)–Sn(1A) 2.7950(13), Si(1)–Si(2) 2.344(3), Si(2)–C(1) 1.878(9), Si(1)–Sn(1)–Sn(1A) 116.47(6).

The molecular structure of distannane **5** in the solid state (Figure 2) is isostructural to that of the respective 1,2-bis[tris(trimethylsilyl)silyl]tetramethyldisilane [48,49] with slightly extended cell parameters. Both compounds crystallize in the triclinic space group *P*-1 and feature perfect *trans*-alignment along the Si–E–E–Si bond arrangement (E = Si, Sn) (Table 2). For the all-silicon molecule we have shown that the conformational preference in the solid state is maintained in solution so that σ-electron conjugation along a chain of six heavy atoms is facile [14]. Sn–Si distances of **5** are with 2.579(3) Å shorter than in **2** reflecting the fact that only one bulky tris(trimethylsilyl)silyl group is attached to a tin atom. The Sn–Sn bond distance of 2.7950(13) is close to that of 2.7930(12) observed for (Me_3_Si)_3_SnSn(SiMe_3_)_3_ [45]. The Si–Sn–Sn bond angle of 119.939(12)° is somewhat increased in comparison to that of the all-silicon analog.

**Figure 3:**
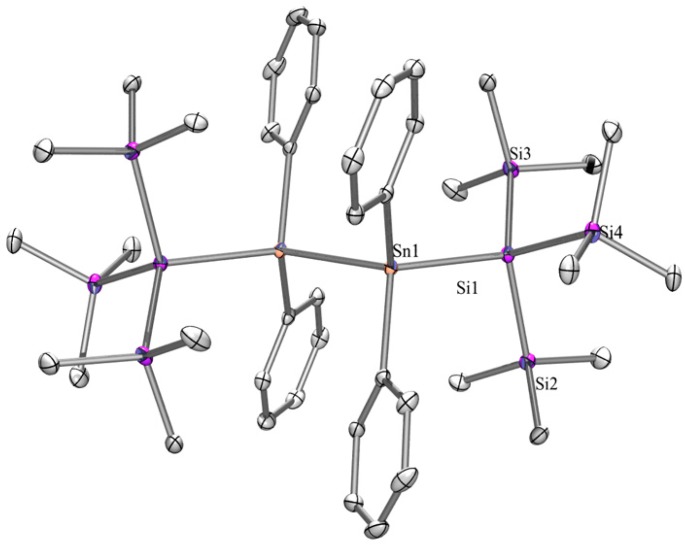
Molecular structure of **6** (thermal ellipsoid plot drawn at the 30% probability level). Non-labeled atoms are generated by symmetry operations. All hydrogen atoms are not shown for clarity (bond lengths in Å, angles in °). Si(1)–Si(2) 2.3476(8), Si(1)–Sn(1) 2.5988(6), Si(2)–C(14) 1.8682(19), Sn(1)–C(1) 2.1603(16), Sn(1)–Sn(1A) 2.8217(7), Si(1)–Sn(1)–Sn(1A) 119.939(12).

Distannane **6** was found to crystallize with one additional benzene molecule in the monoclinic space group *P*2_1_/*n* (Figure 3). Also the structure of **6** contains an inversion center and thus exhibits *trans*-alignment along the Si–Sn–Sn–Si unit (Table 2). Compared to **5** the Sn–Si distances of **6** are slightly elongated to a value of 2.5988(6) Å caused by the increased steric demand of the Ph_2_Sn unit compared to Me_2_Sn. This is also responsible for the elongated Sn–Sn bond distance of 2.8217(7) observed for **6**.

**Figure 4:**
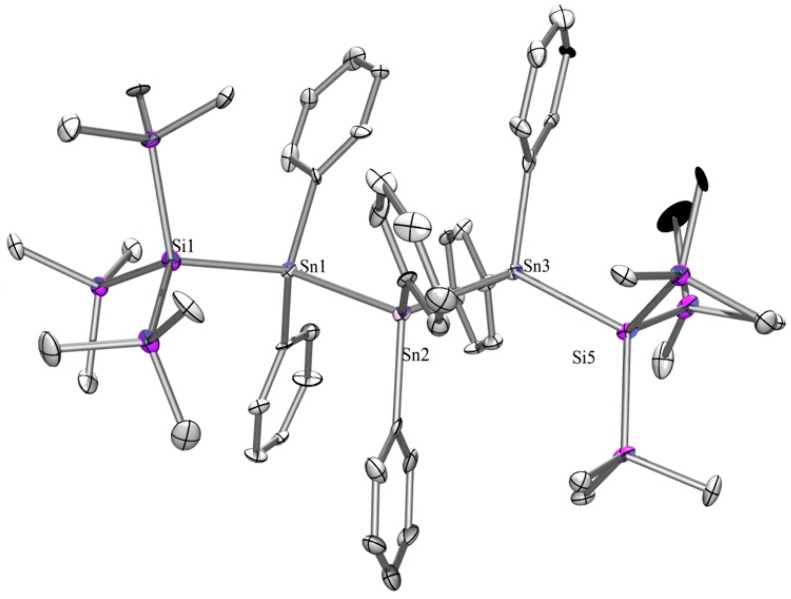
Molecular structure of **6a** (thermal ellipsoid plot drawn at the 30% probability level). All hydrogen atoms are not shown for clarity (bond lengths in Å, angles in °). Sn(1)–C(16) 2.151(14), Sn(1)–Si(1) 2.583(4), Sn(1)–Sn(2) 2.8022(15), Sn(2)–Sn(3) 2.8161(14), Sn(3)–Si(5) 2.608(4), Si(1)–Si(2) 2.358(6), Si(2)–C(1) 1.885(18), Si(1)–Sn(1)–Sn(2) 123.01(11), Sn(1)–Sn(2)–Sn(3) 111.38(4).

The molecular structure of **6a** (Figure 4) in the solid state is asymmetric and a comparably small dihedral angle (148°) on one side of the molecule is observed (Table 2). As expected Sn–Sn bond lengths of **6a** are somewhat shorter than in **6** (2.8022(15) and 2.8161(14) Å) reflecting the diminished interaction between phenyl and trimethylsilyl groups.

**Scheme 3:**
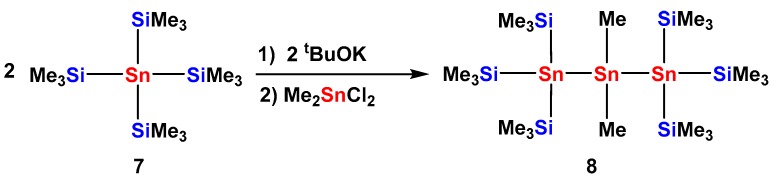
Synthesis of 1,1,1,3,3,3-hexakis(trimethylsilyl)dimethyltristannane **8**.

To study the influence of the exchange of silicon for tin atoms we decided to prepare a structure isostructural to compound **1** but with tris(trimethylsilyl)stannyl end groups. As depicted in Scheme 3 this goal can be achieved by reacting dimethyldichlorostannane with tris(trimethylsilyl)stannyl potassium (obtained easily from tetrakis(trimethylsilyl)stannane (**7**) [34]) yielding compound **8** in 66% yield.

**Figure 5:**
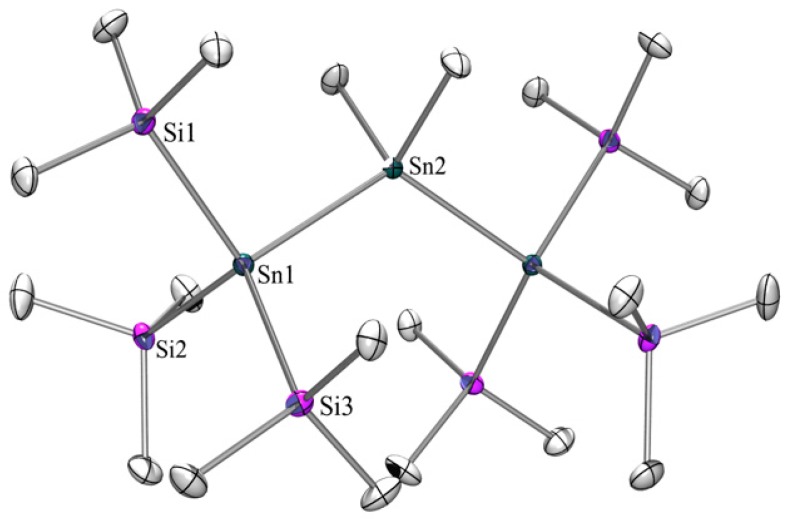
Molecular structure of **8** (thermal ellipsoid plot drawn at the 30% probability level). All hydrogen atoms are not shown for clarity (bond lengths in Å, angles in °). Sn(1)–Si(3) 2.5658(7), Sn(1)–Si(1) 2.5728(8), Sn(1)–Si(2) 2.5758(8), Sn(1)–Sn(2) 2.7871(6), Sn(2)–C(10) 2.179(3), Sn(2)–Sn(1A) 2.7871(6), Si(1)–C(1) 1.876(3), Si(1)–Sn(1)–Sn(2) 105.02(2), Sn(1A)–Sn(2)–Sn(1) 122.946(18).

The structure of compound **8** (Figure 5) again exhibits isostructural properties to the respective all silicon compound Me_2_Si[Si(SiMe_3_)_3_]_2_ [50]. Both compounds crystallize in the monoclinic space group *C*2/*c* with **8** featuring somewhat larger cell axes. The Sn–Sn bond distance of 2.7871(6) Å is close to what was observed for compound **5**. Compared to compound **2** the longer Sn–Sn bonds and the diminished steric demand of the methyl groups at tin larger dihedral angles (Table 2) of 170° are possible.

### 2.2. UV-Vis Spectroscopy

From our studies on the σ-bond electron delocalization of oligosilanes and germaoligosilanes [14,15,16,17,18,19,20,21] we have learned that that tris(trimethylsilyl)silyl terminated methyloligosilanes exibit a strong preference for transoid conformations in the solid state and in solution. Increasing the lengths of the main chain results in bathochromic shifts of the low-energy (σ→σ*) transition indicating extension of the delocalized system. Exchange of selected silicon atoms from the main chain for germanium was found to change the spectroscopic properties only slightly. For a similar exchange of silicon with tin atoms a significantly stronger effect is expected.

**Figure 6:**
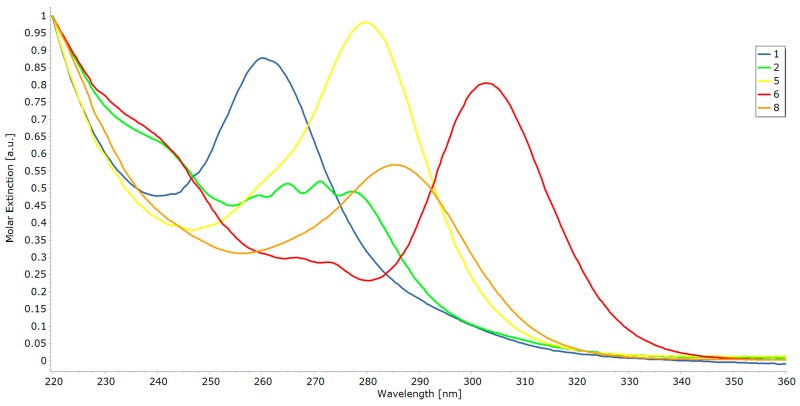
Ultraviolet (UV) spectra of compounds **1**, **2**, **5**, **6**, and **8**.

This expectation is already fulfilled for compound **1**. The low-energy band of its UV spectrum is located at 260 nm (Figure 6) which is 14 nm red shifted compared to the analogous all-silicon compound [(Me_3_Si)_3_Si]_2_SiMe_2_.

Replacement of two additional silicon atoms leads to compound **8** with a central tristannanylene unit. The low-energy band of the UV spectrum of **8** (Figure 6) is located at 286 nm indicating another red shift of 24 nm. Overall, the replacement of three silicon atoms with tin atoms in linear pentasilane unit caused a bathochromic shift of 38 nm.

[(Me_3_Si)_3_Si]_2_(SiMe_2_)_2_, which is the all-silicon analog of compound **5**, exhibits a low-energy band at 253 nm. In contrast to this, the UV spectrum of **5** (Figure 6) with the two central SnMe_2_ units featured the respective band at 280 nm. The formal replacement of two silicon atoms with tin atoms here amounts to a bathochromic shift of 27 nm.

Previous attempts to attach tris(trimethylsilyl)silyl groups to perphenylated silanylene units were not successful, but the stannaoligosilanes of this study allow the study of the influence of attached phenyl groups on the σ-electron delocalization of the oligomers. This can be achieved by comparison of compounds **1** with **2** and compounds **5** with **6**. In addition to the σ→σ* transition bands, the UV spectra of **2** and **6** (Figure 6) also show bands which we assign to π→π* transitions with vibrational fine structure.

Compound **2**, where compared to **1** the two methyl groups on the central tin atom are replaced by phenyl groups, shows a further bathochromic shift of 19 nm for a low-energy band at 279 nm (Figure 6). Still, molecule **2** contains a linear arrangement of five connected heavy atoms where the σ-electron delocalization is supposed to occur. The overall formal replacement of a SiMe_2_ unit in [(Me_3_Si)_3_Si]_2_SiMe_2_ with a SnPh_2_ entity causes a bathochromic shift of the low-energy band of 36 nm. A similar relationship exists between is [(Me_3_Si)_3_Si]_2_(SiMe_2_)_2_ (λ_max_ = 253 nm) and compound **6** which extends its low-energy band to 303 nm. The formal exchange of a tetramethyldisilanylene unit with a tetraphenyldistannanylene in this case causes a 50 nm bathochromic shift.

## 3. Materials and Methods

### Experimental Section

All reactions involving air-sensitive compounds were carried out under an atmosphere of dry nitrogen or argon using either Schlenk techniques or a glove box. Solvents were dried using a column solvent purification system [51]. Potassium *tert*-butanolate was purchased exclusively from MERCK. Dichlorodiphenylstannane, dichlorodimethylstannane, hexaphenyldistannane (**4**) and all other chemicals were obtained from different suppliers and were used without further purification. Tris(trimethylsilyl)silyl potassium [35,36], and tetrakis(trimethylsilyl)stannane (**7**) [52] have been prepared following published procedures.

^1^H- (300 MHz), ^13^C- (75.4 MHz), ^119^Sn- (111.8 MHz), and ^29^Si- (59.3 MHz) NMR spectra were recorded on a Varian Unity INOVA 300 spectrometer (Varian, Palo Alto, CA, USA). If not noted otherwise for all samples the used solvent was C_6_D_6_ or in case of reaction samples they were measured with a D_2_O capillary in order to provide an external lock frequency signal. To compensate for the low isotopic abundance of ^29^Si the INEPT pulse sequence was used for the amplification of the signal [53,54]. Elementary analysis was carried using a Heraeus VARIO ELEMENTAR EL apparatus (Heraeus, Hanau, Germany). UV spectra were measured on a Perkin Elmer Lambda 35 spectrometer (Perkin-Elmer Corp., Waltham, MA, USA) using spectroscopy grade pentane as solvent. Spectra plotting was done using Spectragryph 1.07 [55].

X-ray structure determination: For X-ray structure analyses the crystals were mounted onto the tip of glass fibers, and data collection was performed with a BRUKER-AXS SMART APEX CCD diffractometer (Bruker-AXS, Madison, WI, USA) using graphite-monochromated Mo Kα radiation (0.71073 Å). The data were reduced to F^2^_0_ and corrected for absorption effects with SAINT [56] and SADABS [57,58], respectively. Structures were solved by direct methods and refined by full-matrix least-squares method (SHELXL97 and SHELX2013) [59,60]. All non-hydrogen atoms were refined with anisotropic displacement parameters. Hydrogen atoms were placed in calculated positions to correspond to standard bond lengths and angles.

Crystallographic data for the structures of compounds **2**, **5**, **6**, **6a**, and **8** reported in this paper have been deposited with the Cambridge Crystallographic Data Center as supplementary publication no. CCDC 1585436 (**2**), 1585433 (**5**), 1585432 (**6**), 1585434 (**6a**), and 1585435 (**8**). Copies of data can be obtained free of charge at: http://www.ccdc.cam.ac.uk/products/csd/request/. Figures of solid state molecular structures were generated using Ortep-3 as implemented in WINGX [61] and rendered using POV-Ray 3.6 [62].

*Dimethyldiphenylstannane.* A solution of PhMgBr in Et_2_O (prepared from Mg (466 mg, 19.2 mmol) and bromobenzene (2986 mg, 19.2 mmol) was slowly added to dichlorodimethylstannane (2057 mg, 9.4 mmol) in THF (20 mL) at 0 °C. The mixture was stirred for 12 h before saturated NH_4_Cl solution (30 mL) was added. The organic phase was separated, reduced in volume, and then passed through a pad of silica gel. After evaporation of solvent dimethyldiphenylstannane (1829 mg, 64%) was obtained as a colorless oil. NMR spectroscopic data in accordance with published values [63]: ^119^Sn-NMR (δ in ppm): −60.

*Chlorodimethylphenylstannane.* To a solution of dimethyldiphenylstannane (1829 mg, 5.3 mmol) in Et_2_O (25 mL) HCl (1.2 equiv., 1 M solution in diethyl ether) was added at 0 °C. The mixture was allowed to come to r.t. and then stirred for 12 h before saturated NH_4_Cl solution (10 mL) was added. The layers were separated, the organic layer dried over anhydrous Na_2_SO_4_ and the solvent removed. Chlorodimethylphenylstannane (851 mg, 61%) was obtained as a colorless oil and was used without further purification. NMR spectroscopic data in accordance with published values [63]: ^119^Sn-NMR (δ in ppm): 91.

*1,1-Bis[tris(trimethylsilyl)silyl]dimethylstannane* (**1**)*.* A solution of tris(trimethylsilyl)silyl potassium (prepared from tetrakis(trimethylsilyl)silane (750 mg, 2.3 mmol) and *t*BuOK (284 mg, 2.4 mmol) in DME (8 mL) was added to dichlorodimethylstannane (262 mg, 1.2 mmol) in THF (5 mL) very slowly at r.t. and then stirred for 12 h. To the reaction mixture pentane (10 mL), THF (10 mL), and 2M H_2_SO_4_ (10 mL) were added, the phases separated, the organic phase dried with anhydrous Na_2_SO_4_ and the solvent removed. Compound **1** was obtained as colorless crystals (668 mg, 86%) by crystallization from a pentane/acetone 1:1 solution at r.t. m.p.: 85–95 °C. ^1^H-NMR (δ in ppm): 0.34 (s, 54H, SiMe_3_), 0.26 (s, 6H, SnMe_2_). ^29^Si-NMR (δ in ppm): −7.7 (SiMe_3_), −123.7 (Si_q_). ^119^Sn-NMR (δ in ppm): −176. Anal. calcd. for C_20_H_60_Si_8_Sn (644.09): C 37.30, H 9.39. Found: C 37.20, H 9.32. UV absorption: λ_1_ = 209 nm (ε_1_ = 1.7 × 10^4^ M^−1^ cm^−1^), λ_2_ = 260 nm (ε_2_ = 2.4 × 10^4^ M^−1^ cm^−1^).

*1,1-Bis[tris(trimethylsilyl)silyl]diphenylstannane* (**2**). Same procedure as for **1** using: dichlorodiphenylstannane (446 mg, 1.3 mmol), tetrakis(trimethylsilyl)silane (757 mg, 2.4 mmol) and *t*BuOK (312 mg, 2.5 mmol). Colorless crystalline **2** (613 mg, 61%) was obtained. m.p.: 143–145 °C. ^1^H-NMR (δ in ppm): 7.79 (m, 4H), 7.09 (m, 6H), 0.18 (s, 54H, SiMe_3_). ^13^C-NMR (δ in ppm): 142.7, 138.4, 127.9, 127.7, 3.5. ^29^Si-NMR (δ in ppm): −9.9 (SiMe_3_), −133.9 (Si_q_). ^119^Sn-NMR (δ in ppm): −174 (^1^*J*_Sn–C_ = 264 Hz, ^1^*J*_Si–Sn_ = 182 Hz, ^2^*J*_Si–Sn_ = 39 Hz). Anal. calcd. for C_28_H_64_Si_8_Sn (768.23): C 46.90, H 8.40. Found: C 44.65, H 8.33. UV absorption: λ_1_ = 209 nm (ε_1_ = 1.7 × 10^4^ M^−1^ cm^−1^), λ_2_ = 260 nm (ε_2_ = 2.4 × 10^4^ M^−1^ cm^−1^).

*1,2-Diphenyltetramethyldistannane* (**3**). To chlorodimethylphenylstannane (851 mg, 3.3 mmol) in THF (8 mL) metallic sodium (75 mg, 3.3 mmol) was added and stirred at r.t. for 48 h. A saturated NH_4_Cl solution (10 mL) was added and extracted with Et_2_O. The organic layer was dried with over anhydrous Na_2_SO_4_ and then the solvent removed. Compound **3** (578 mg, 48%) was obtained as a colorless oil with spectroscopic values in accordance to published data [46,47]. ^119^Sn-NMR (δ in ppm): −118.

*1,2-Bis[tris(trimethylsilyl)silyl]tetramethyldistannane* (**5**). To a solution of **3** (578 mg, 1.3 mmol) in CH_2_Cl_2_ (10 mL) triflic acid (384 mg, 2.6 mmol) was added dropwise at −40 °C. The reaction mixture was allowed to come to r.t. and was stirred for additional 12 h. (The clear solution was checked by ^119^Sn-NMR (δ in ppm): 107 ppm) [64]. The solvent was removed and the residue dissolved in toluene (10 mL) followed by the dropwise addition of tris(trimethylsilyl)silyl potassium [prepared from tetrakis(trimethylsilyl)silane (821 mg, 2.6 mmol) and *t*BuOK (316 mg, 2.8 mmol) in DME (8 mL). The solvent was removed and the residue solved in toluene (10 mL)]. The reaction solution turns yellow and then dark orange. Stirring was continued for 12 h. The work up procedure was done according to the procedure for **1**. After recrystallization from CH_2_Cl_2_ colorless crystalline **5** (483 mg, 48%) was obtained. m.p.: 222–225 °C. ^1^H-NMR (δ in ppm, CDCl_3_): 0.38 (s, ^2^*J*_H–Sn_ = 40.5 Hz, ^3^*J*_H–Sn_ = 19.6 Hz, 12H, SnMe_2_), 0.22 (s, *J* = 12.3Hz, *J* = 10.2 Hz, *J* = 6.5 Hz, 54H, SiMe_3_). ^13^C-NMR (δ in ppm, CDCl_3_): 3.1 (SiMe_3_), −8.7 (^1^*J*_C–Sn_ = 36.8 Hz, SnMe_2_). ^29^Si-NMR (δ in ppm, CDCl_3_): −7.8 (SiMe_3_), −128.7 (Si_q_). ^119^Sn-NMR (δ in ppm): −68. Anal. calcd. for C_22_H_66_Si_8_Sn (792.87): C 33.33, H 8.39. Found: C 33.21, H 8.29. UV absorption: λ_1_ = 209 nm (ε_1_ = 1.7 × 10^4^ M^−1^ cm^−1^), λ_2_ = 260 nm (ε_2_ = 2.4 × 10^4^ M^−1^ cm^−1^).

*1,2-Bis[tris(trimethylsilyl)silyl]tetraphenyldistannane* (**6**). The same procedure as for **1** was applied using hexaphenyldistannane (2000 mg, 2.8 mmol), triflic acid (858 mg, 5.7 mmol) [the addition of triflic acid was done at r.t. and addition of triflate was checked by ^119^Sn-NMR (δ in ppm): −100.2 ppm] [64], tetrakis(trimethylsilyl)silane (1832 mg, 5.7 mmol) and *t*BuOK (705 mg, 6.3 mmol). The addition of tris(trimethylsilyl) potassium and the following stirring was done at 40 °C. The pale yellow solid was recrystallized from pentane/THF at −20 °C to yield colorless crystalline **6** (619 mg, 33%). In addition to **6**, a few crystals of 1,3-bis[tris(trimethylsilyl)silyl]hexaphenyltristannane (**6a**) were also obtained. **6**: m.p.: 226–228 °C. ^1^H-NMR (δ in ppm): 7.75 (m, 4H), 7.41 (m, 2H), 7.02 (m, 4H), 0.26 (s, 54H, SiMe_3_). ^13^C-NMR (δ in ppm, THF-*d*_8_): 143.6, 140.9, 130.4, 130.3, 4.7 (SiMe_3_). ^29^Si-NMR (δ in ppm): −8.4 (SiMe_3_), −131.8 (Si_q_). ^119^Sn-NMR (δ in ppm): −114. Anal. calcd. for C_42_H_74_Si_8_Sn_2_ (1041.15): C 48.45, H 7.16. Found: C 48.39, H 7.09. UV absorption: λ_1_ = 209 nm (ε_1_ = 1.7 × 10^4^ M^−1^ cm^−1^), λ_2_ = 260 nm (ε_2_ = 2.4 × 10^4^ M^−1^ cm^−1^).

*1,1,1,3,3,3-Hexakis(trimethylsilyl)dimethyltristannane* (**8**). A solution of tetrakis(trimethylsilyl)-stannane (397 mg, 0.97 mmol) in DME (5 mL) was treated with *t*BuOK (112 mg, 0.99 mmol) in DME at r.t. After 12 h the solvent was removed and the residue dissolved with toluene (5 mL). This solution was added dropwise to dichlorodimethylstannane (106 mg, 0.48 mmol) in THF (5 mL) and stirred for 12 h. A saturated NH_4_Cl solution (5 mL) was added, the phases separated, the organic phase dried with anhydrous Na_2_SO_4_ and the solvent removed. After recrystallization from CH_2_Cl_2_ colorless crystalline **8** (272 mg, 66%) was obtained. m.p.: 134–136 °C. ^1^H-NMR (δ in ppm, CDCl_3_): 0.32 (s, 18H, SiMe_3_), 0.29 (s, 6H, SnMe_2_). ^13^C-NMR (δ in ppm, CDCl_3_): 4.8 (Sn(SiMe_3_)_3_), −7.1 (SnMe_2_). ^29^Si-NMR (δ in ppm, D_2_O capillary): −7.1 (^1^*J*_117/119Sn–Si_ = 324 Hz, 310 Hz, SiMe_3_). ^119^Sn-NMR (δ in ppm, THF-*d*_8_): −277 (SnMe_2_), −464 (Sn(SiMe_3_)_3_). Anal. calcd. for C_20_H_60_Si_6_Sn_3_ (825.34): C 29.11, H 7.33. Found: C 29.02, H 7.21. UV absorption: λ_1_ = 209 nm (ε_1_ = 1.7 × 10^4^ M^−1^ cm^−1^), λ_2_ = 260 nm (ε_2_ = 2.4 × 10^4^ M^−1^ cm^−1^).

## 4. Conclusions

With the intention to study the influence of incorporated heavier group 14 element atoms, a number of stannaoligosilanes were synthesized. The UV absorption properties of these were compared to those of previously investigated oligosilanes of equal chain lengths and substituent pattern. Replacing one dimethylsilylene group in a pentasilane by a dimethylstannylene unit was found to cause a bathochromic shift of the low-energy band (corresponding to HOMO-LUMO transition) (λ_max_ = 260 nm) of 14 nm. By introduction of a diphenylstannylene group the bathochromic shift is raised to 33 nm. Keeping the dimethylstannylene unit and exchanging the two adjacent silicon atoms by tin shifts the respective band (λ = 286 nm) some 26 nm to the red.

These results clearly show that by deliberate exchange of silicon for tin and switching to phenyl substituents, the absorption properties and the degree of σ-bond electron delocalization can be tuned over a large spectral range.

## Figures and Tables

**Table 1:** NMR spectroscopic characterization of **1**, **2**, **5**, **6**, and **8**.

Compound	^29^Si (*Si*Me_3_)	^29^Si *Si*(EMe_3_)	^119^Sn
**1**	−7.7	−123.7	−176
**2**	−9.9	−133.9	−174
**5**	−7.8	−128.7	−68
**6**	−8.4	−131.8	−114
**8**	−7.1	-	−277 (SnMe), −464 (SnSi)

**Table 2:** Dihedral angles of the Si_1–2_–(Sn)_1–3_–Si_1–2_ framework of compounds **2**, **5**, **6**, **6a**, and **8**.

Dihedral Angles *ω* [°] Determined by X-ray Crystallography					
Compound	*ω*_1_	*ω*_2_	*ω*_3_	*ω*_4_	Conformation ^1^
**2**	153	143			DD
**5**	159	180	159		TAT
**6**	160	180	160		TAT
**6a**	162	162	161	148	TTTD
**8**	170	170			TT

^1^ A = anti, T = transoid, D = deviant.

## Supplementary Materials

The following are available online, Table S1: Crystallographic information for compounds **2**, **5**, **6**, **6a**, and **8**.
