# Supplementary material for: Incorporating Methyl and Phenyl Substituted Stannylene Units into Oligosilanes. The Influence on Optical Absorption Properties
Source: Molecules. 2017 Dec 12;22(12):2212. doi: 10.3390/molecules22122212 (PMC6149905; doi:10.3390/molecules22122212)
Supplement: Supplementary file 1 [file molecules-22-02212-s001.pdf]

Supporting Info for

Incorporating Methyl and Phenyl Substituted Stannylene Units into Oligosilanes. The Influence on Optical Absorption Properties

Filippo Stella [1], Christoph Marschner [1,*] and Judith Baumgartner [2,*]

[1] Institute for Inorganic Chemistry, Graz University of Technology, Stremayrgasse 9, 8010 Graz, Austria; christoph.marschner@tugraz.at

[2] Institute for Chemistry, Karl-Franzens-University Graz, Stremayrgasse 9, 8010 Graz, Austria

Table S1: Crystallographic data for compounds **2**, **5**, **6**, **6a** and **8**

	**2**	**5**	**6**	**6a**	**8**
Empirical formula	$C_{30}H_{64}Si_8Sn$	$C_{22}H_{54}Si_8Sn_2$	$C_{48}H_{80}Si_8Sn_2$	$C_{54}H_{84}Si_8Sn_3$	$C_{20}H_{54}Si_6Sn_3$
M_w	768.22	780.75	1119.22	1314.00	819.24
Temperature [K]	100(2)	100(2)	100(2)	100(2)	100(2)
Size [mm]	0.44×0.25×0.19	0.48×0.34×0.12	0.26×0.22×0.18	0.32×0.28×0.28	0.44×0.41×0.10
Crystal system	monoclinic	triclinic	monoclinic	monoclinic	monoclinic
Space group	P2(1)/c	P-1	P2(1)/n	P2(1)/n	C2/c
a [Å]	10.605(2)	9.070(2)	13.099(3)	16.707(3)	17.304(4)
b [Å]	18.310(4)	9.160(2)	16.486(3)	25.803(5)	9.390(2)
c [Å]	22.826(5)	14.035(3)	13.735(3)	17.153(3)	24.871(5)
α [°]	90	92.14(3)	90	90	90
β [°]	91.74(3)	103.36(3)	98.99(3)	95.61(3)	105.54(3)
γ [°]	90	113.61(3)	90	90	90
V [$Å^3$]	4430(2)	1028(2)	2929(2)	6819(2)	3894(2)
Z	4	1	2	4	4
ρ_{calc} [gcm^{-3}]	1.152	1.261	1.269	1.280	1.398
Absorption coefficient [mm^{-1}]	0.810	1.457	1.045	1.260	2.101
F(000)	1624	398	1160	2680	1632
θ range	1.43<θ<26.37	1.51<θ<26.33	1.94<θ<26.37	1.44<θ<25.00	1.70<θ<26.36
Reflections collected/unique	28319/8939	7977/4064	23090/5950	37389/11449	15120/3956
Completeness to θ [%]	98.6	96.7	99.2	95.4	99.4
Data/restraints/parameters	8939/0/370	4064/0/154	5950/0/271	11449/24/604	3956/0/141
Goodness of fit on F^2	1.01	1.09	1.07	1.13	1.06
Final R indices [I>2σ(I)]	R1=0.029, *w*R2=0.069	R1=0.060, *w*R2=0.168	R1=0.021, *w*R2=0.053	R1=0.106, *w*R2=0.239	R1=0.022, *w*R2=0.058
R indices (all data)	R1=0.036, *w*R2=0.071	R1=0.061, *w*R2=0.168	R1=0.022, *w*R2=0.054	R1=0.148, *w*R2=0.280	R1=0.023, *w*R2=0.059
Largest diff. Peak/hole [e^-/ $Å^3$]	0.73/-0.26	3.75/-1.39	0.41/-0.56	2.97/-2.48	0.85/-0.86
